# Hyperactive gp130/STAT3‐driven gastric tumourigenesis promotes submucosal tertiary lymphoid structure development

**DOI:** 10.1002/ijc.31298

**Published:** 2018-02-19

**Authors:** David G. Hill, Liang Yu, Hugh Gao, Jesse J. Balic, Alison West, Hiroko Oshima, Louise McLeod, Masanobu Oshima, Awen Gallimore, Kimberley D'Costa, Prithi S. Bhathal, William Sievert, Richard L. Ferrero, Brendan J. Jenkins, Gareth W. Jones

**Affiliations:** ^1^ Division of Infection and Immunity Systems Immunity Research Institute, School of Medicine, Cardiff University Cardiff Wales United Kingdom; ^2^ Centre for Innate Immunity and Infectious Diseases Hudson Institute of Medical Research Clayton VIC Australia; ^3^ Department of Molecular and Translational Science, Faculty of Medicine, Nursing and Health Sciences Monash University Clayton VIC Australia; ^4^ Division of Genetics Cancer Research Institute, Kanazawa University Kanazawa Japan; ^5^ Department of Medicine Monash Medical Centre, Monash University Clayton VIC Australia; ^6^ Biomedicine Discovery Institute, Department of Microbiology Monash University Clayton VIC Australia

**Keywords:** gastric cancer, tertiary lymphoid structures, ectopic lymphoid structures, STAT3, interleukin‐17

## Abstract

Tertiary lymphoid structures (TLSs) display phenotypic and functional characteristics of secondary lymphoid organs, and often develop in tissues affected by chronic inflammation, as well as in certain inflammation‐associated cancers where they are prognostic of improved patient survival. However, the mechanisms that govern the development of tumour‐associated TLSs remain ill‐defined. Here, we observed tumour‐associated TLSs in a preclinical mouse model (*gp130* ^F/F^) of gastric cancer, where tumourigenesis is dependent on hyperactive STAT3 signalling through the common IL‐6 family signalling receptor, gp130. Gastric tumourigenesis was associated with the development of B and T cell‐rich submucosal lymphoid aggregates, containing CD21^+^ cellular networks and high endothelial venules. Temporally, TLS formation coincided with the development of gastric adenomas and induction of homeostatic chemokines including *Cxcl13*, *Ccl19* and *Ccl21*. Reflecting the requirement of gp130‐driven STAT3 signalling for gastric tumourigenesis, submucosal TLS development was also STAT3‐dependent, but independent of the cytokine IL‐17 which has been linked with lymphoid neogenesis in chronic inflammation and autoimmunity. Interestingly, upregulated lymphoid chemokine expression and TLS formation were also observed in a chronic gastritis model induced by *Helicobacter felis* infection. Tumour‐associated TLSs were also observed in patients with intestinal‐type gastric cancer, and a gene signature linked with TLS development in *gp130* ^F/F^ mice was associated with advanced clinical disease, but was not prognostic of patient survival. Collectively, our *in vivo* data reveal that hyperactive gp130‐STAT3 signalling closely links gastric tumourigenesis with lymphoid neogenesis, and while a TLS gene signature was associated with advanced gastric cancer in patients, it did not indicate a favourable prognosis.

## Abstract 2

**What's new?**

Tertiary lymphoid structures (TLSs) develop in chronically inflamed tissues, and have been associated with improved survival in certain cancer patients. Here, the authors examined mechanisms governing the development of submucosal TLSs in the gp130F/F mouse model of gastric cancer and in patients afflicted with intestinal‐type disease. TLS formation was observed both mice and patients but a TLS gene signature identified in mice did not predict improved patient survival, pointing to need for more research into TLSs and gastric cancer prognosis.

## Introduction

Gastric cancer (GC) is the third most lethal cancer worldwide and affects over half a million people per year.^1^ Chronic infection with *Helicobacter pylori* accounts for >75% of GC, and a well‐established causal relationship exists between *H. pylori‐*triggered immune dysregulation and GC progression.^1^ Thus, GC represents a growing number of cancers including colon, lung, liver and prostate where chronic inflammation is a prominent feature. Two major contributing factors to the poor overall survival rate (<25%) in GC are late diagnosis and the limited effectiveness of current treatment options. These factors are compounded by the heterogeneity of the host mucosal immune response and the diverse molecular complexity of gastric adenocarcinomas.^2^, ^3^ Thus, intra‐ and inter‐tumour heterogeneity present challenges to identifying robust biomarkers, that can aid early detection of disease and inform personalised treatment strategies for GC patients.

Persistent non‐resolving gastritis, associated with defective anti‐inflammatory control and a failure to eradicate microbial pathogens, provides a microenvironment that supports accelerated tumour progression, invasion of surrounding tissues, angiogenesis and metastasis.^1^ However, the mechanisms that link chronic inflammation to the development and progression of GC are ill‐defined. Tertiary lymphoid structures (TLSs; also called ectopic lymphoid structures, ELSs) are inducible lymphoid aggregates that phenotypically and functionally resemble secondary lymphoid organs.^4^ TLSs can develop in tissues where persistent inflammation is present, and a correlation between high TLS densities and prolonged patient survival has been reported for several cancers including breast,^5^, ^6^ lung,^7^, ^8^ melanoma,^9^ pancreatic^10^ and colorectal.^11^, ^12^ TLS gene signatures, that include homeostatic chemokines such as *CXCL13*, *CCL19* and *CCL21*, have the potential to represent prognostic indicators of cancer progression in melanoma^13^ and colorectal cancer.^14^, ^15^ Several studies propose that tumour‐associated TLSs provide a local environment for establishing and maintaining anti‐tumour immunity.^4^, ^12^ For example, clonal expansion of interferon‐(IFN)‐γ producing T cells reactive to tumour antigens, which inhibit tumour growth, have been observed in experimental mouse melanoma.^16^, ^17^ Improved patient survival in non‐small‐cell lung carcinoma has also been linked with TLSs, where follicular B cells and plasma cells show antibody specificity to tumour antigens.^8^ However, TLS involvement and activity may only have prognostic value in certain stages of tumour development or for certain types of cancer.^11^, ^18^, ^19^ Thus, greater mechanistic insights are required to establish the link between TLS formation, tumour progression and clinical outcome.

Recently, tumour‐associated TLSs were described in GC, and histological evidence of a coordinated local B cell (CD20^+^) and T helper (Th)1 (T‐bet^+^) response was associated with improved relapse‐free survival.^20^ Therefore, TLSs represent attractive immunomodulatory targets for improving anti‐tumour immunity.^4^, ^12^ Members of the IL‐6 cytokine family, including IL‐6 and IL‐27, and other cytokines such as IL‐21 that activate the oncogenic latent transcription factor STAT3, have emerged as regulators of TLS development, maintenance and activity.^21^, ^22^, ^23^ We have previously generated *gp130* ^F/F^ mice, which spontaneously develop inflammation‐associated intestinal‐type gastric tumours.^24^, ^25^ At the molecular level, these mice contain a knock‐in phenylalanine mutation at tyrosine 757 in the cytoplasmic domain of gp130, the common IL‐6 family signalling receptor, disrupts the negative feedback imposed on gp130 signalling by suppressor of cytokine signalling (SOCS)3. Consequently, gastric tumourigenesis in *gp130* ^F/F^ mice is driven by STAT3 hyperactivation in response to the IL‐6 family cytokine, IL‐11.^25^, ^26^ The clinical relevance of the *gp130* ^F/F^ mouse model is illustrated by the observation that human GC tumours are associated with high IL‐11 expression and STAT3 hyperactivation.^27^, ^28^ Using the *gp130* ^F/F^ GC model, we reveal that the gp130/STAT3 signalling axis drives TLS development concomitant with a lymphoid chemokine signature, which although is associated with advanced GC in patients, does not indicate a favourable prognosis.

## Materials and Methods

### Patient samples

Antral gastric biopsies were collected from GC patients enrolled at Monash Medical Centre (*n* = 6) undergoing surgical resection. In addition, tissue biopsies were collected by endoscopy (performed at Monash Medical Centre) from the gastric antrum and corpus regions of individuals displaying gastritis with intestinal metaplasia (*n* = 10). Biopsies were stored in 10% formalin for histopathological assessment and *H. pylori* status using the updated version of the Sydney System.^29^, ^30^ Full, informed patient consent was obtained and biopsy collections were approved by the Monash Health and Monash University Human Research Ethics Committees.

### Mice

The *gp130* ^F/F^ mice and their compound mutant derivatives either heterozygous for *Stat3* (*gp130* ^F/F^:*Stat3*±) or deficient in *Il17a* (*gp130* ^F/F^:*Il17a* ^−^^*/*^^−^), have been described previously.^24^, ^26^, ^30^ Where appropriate, experiments used age‐matched wild‐type (WT) mice. All mice were on a mixed C57BL/6 × 129/Sv background and were housed under specific pathogen‐free conditions. Experiments were approved by the Monash Medical Centre ‘A’ Monash University Animal Ethics Committee.

### Helicobacter infection model

*Helicobacter felis* suspensions for mouse inoculation were prepared by harvesting bacteria from horse blood agar plates using Brain Heart Infusion broth (BHI; Oxoid).^31^ Six‐ to eight‐week‐old SPF wild‐type 129Sv × C57BL/6 mice were intragastrically administered a single 100 µL aliquot of either control BHI, or approximately 10^7^ *H. felis* bacteria, using polyethylene catheters.^31^ Infection was confirmed at 16 weeks by polymerase chain reaction (PCR) amplification of the *H. felis flaB* gene (see Supporting Information Table S1).

### Histology and immunohistochemistry

Formalin‐fixed paraffin‐embedded mouse stomachs were sectioned and stained with H&E for histology as described previously.^30^ Submucosal lymphoid aggregates were characterised in serial sections by immunohistochemistry, using antibodies specific to mouse B220 (BD Biosciences, UK), CD3 (Dako, Agilent Technologies, UK), podoplanin (BioLegend, UK), p‐Y^705^STAT3 (Cell Signaling Technology, from New England BioLabs, UK), CD21 (Santa Cruz Biotechnology, Germany), CXCL13 (R&D Systems, UK), PNAd (MECA‐79), Bcl‐6 (Santa Cruz Biotechnology) and Ki67 (Abcam, UK). Lymphoid aggregates in human GC biopsies were detected using antibodies against CD20 and CD3 (both from Dako). Antigen unmasking and blockade of endogenous peroxidase activity, endogenous biotin and non‐specific antibody binding was performed as described previously.^22^ Antibody labelling was detected using biotinylated secondary antibodies (Dako), the Vectastain ABC kit and diaminobenzidine chromagen (DAB) (Vector Laboratories, UK). For the staining of multiple antigens in the same tissue section, DAB chromagen was used in conjunction with VIP (Vector Laboratories). For the detection of germinal centres, sections were first incubated with biotinylated peanut agglutinin before staining using the Vectastain ABC kit and DAB chromagen (all from Vector Laboratories). The Leica QWin microscope imaging software was used to quantify positive staining.

### RNA isolation and quantitative real‐time PCR (qPCR)

Total RNA was recovered from snap‐frozen gastric antrum and tumour tissues using TRIzol reagent (Thermo Fisher Scientific, Australia) followed by DNase treatment. The Transcriptor High Fidelity cDNA Synthesis Kit (Roche, Australia) was used to prepare cDNA. For RNA extraction from laser microdissected gastric tissues, stroma samples were firstly collected from OCT‐embedded frozen sections stained with toluidine blue using laser microdissection (Leica Microsystems, Japan), following which total RNA was extracted using the miRNeasy microkit (Qiagen, Japan) and then reverse transcribed with the PrimeScript RT Reagent Kit (Takara, Japan). SYBR Green (Invitrogen, Thermo Fisher Scientific, Australia) detection of transcript amplification was performed on the 7900HT Fast RT‐PCR System (Applied Biosystems, Thermo Fisher Scientific, Australia). Gene expression analysis was performed using the Sequence Detection System Version 2.3 software (Applied Biosystems). Primer sequences for amplification of target gene transcripts and housekeeping *18S rRNA* are presented in Supporting Information Table S1.

### Bioinformatic analysis of intestinal‐type GC patient cohorts

Clinical information and mRNA sequencing data were obtained from The Cancer Genome Atlas (TCGA) data portal (https://portal.gdc.cancer.gov/projects/TCGA-STAD). Alignment of sample identifiers yielded 389 tumour cases, with 176 intestinal‐type GC patients. Matched tumour and non‐tumour tissues were available for 35 GC patients. Clinicopathological features and patient demographics are presented in Supporting Information Table S2. We used reads per kilobase of exon per million mapped reads (RPKM)^32^ to represent expression levels. Single‐sample Gene Set Enrichment Analysis (ssGSEA)^33^ was used to investigate a three‐gene signature (*CXCL13*, *CCL19*, *CCL21*) linked with TLS development in *gp130* ^F/F^ mice. This data was also validated in an independent cohort of intestinal‐type GC patients from the Asian Cancer Research Group (ACRG; GEO accession number GSE62254).^34^

### Statistics

Statistical analysis was performed using GraphPad Prism software. For normally distributed data, statistical significance between groups was determined using one‐way ANOVA with Tukey's multiple comparison's test. Where genotypes were compared across multiple time points, a two‐way analysis of variance (ANOVA) was performed. For data that was not normally distributed, a non‐parametric Mann–Whitney *U‐*test was performed. A *p* < 0.05 was considered statistically significant. Graphs are presented as mean ± standard error of the mean (SEM).

## Results

### Gastric tumourigenesis in *gp130* ^F/F^ mice is associated with submucosal TLS development

**Figure 1:**
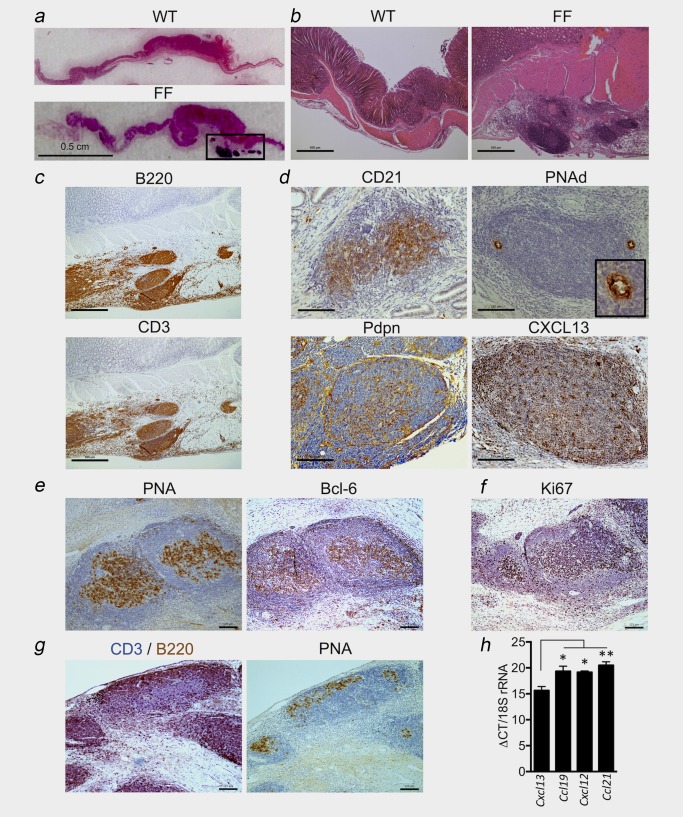
Gastric tumourigenesis in *gp130* ^F/F^ mice is associated with TLS development. (*a* and *b*) Representative haematoxylin and eosin (H&E) stained cross‐sections through the antral gastric region of 6‐month‐old *gp130^+/+^* (WT) and *gp130* ^F/F^ (FF) mice. Boxed region in (*a*) indicates cellular aggregates in the tumoural submucosa of *gp130* ^F/F^ mice. (*c*) Representative immunohistochemistry of serial sequential sections showing co‐localisation of B220 (B cells) and CD3 (T cells) at lymphoid aggregates in 6‐month‐old *gp130* ^F/F^ mice. (*d*) Representative immunohistochemistry of CD21, PNAd^+^ HEV with high‐power image inset, podoplanin (Pdpn) and CXCL13 at lymphoid aggregates in 6‐month‐old *gp130* ^F/F^ mice. (*e* and *f*) Representative immunohistochemistry of peanut agglutinin (PNA) and Bcl‐6 in sequential sections (*e*) and the proliferative marker Ki67 (*f*) at germinal centres in 6‐month‐old *gp130* ^F/F^ mice. (*g*) Dual staining for CD3 (purple) and B220 (brown) (left image) and PNA (right image) in sequential sections at lymphoid aggregates in 6‐month‐old *gp130* ^F/F^ mice. PNA staining at germinal centres is seen in lymphoid aggregates displaying segregated B and T cell zones, and in less organised cellular aggregates. (*h*) Quantitative RT‐PCR (qPCR) analyses of *Cxcl13*, *Ccl19*, *Cxcl12* and *Ccl21* normalised to 18S rRNA and displayed as median qPCR cycle threshold (ΔCT) in RNA purified from laser capture microdissected submucosal lymphoid aggregates in 6‐month‐old *gp130* ^F/F^ mice (*n* = 3). *p < 0.05; **p < 0.01. Scale bars: (*a*) 0.5 cm; (*b* and *c*) 500 µm; (*d*–*g*) 125 µm. [Color figure can be viewed at http://wileyonlinelibrary.com]

The spontaneous development of gastric antrum adenomas in *gp130* ^F/F^ mice is associated with inflammatory infiltrates and accumulations of large extra‐tumoural inflammatory aggregates in the gastric submucosa, the latter of which are absent in age‐matched 6‐month‐old control WT (*gp130* ^+/+^) mice^24^, ^25^, ^26^ (Figs. 1 *a* and 1 *b*). We initially employed immunohistochemistry to assess whether these submucosal inflammatory aggregates were characteristic of TLSs. Indeed, consistent with the cellular features of tumour‐associated TLSs, immunohistochemistry revealed a typical pattern of lymphoid organisation, with a co‐localisation of B220^+^ B cells and CD3^+^ T cells within cellular aggregates (Fig. 1 *c*). Further characterisation identified the presence of cellular CD21^+^ networks and peripheral lymph node addressin (PNAd)^+^ high endothelial venules (HEV) (Fig. 1 *d*). TLSs also contained podoplanin (Pdpn; also called gp38) expressing cells, marking the presence of stromal cells required for lymphoid organ development, and the support of lymphocyte migration and antigen presentation^4^, ^35^ (Fig. 1 *d*). The presence of CXCL13‐expressing cells, a homeostatic chemokine involved in TLS and secondary lymphoid organ development, was also observed (Fig. 1 *d*). Consistent with the presence of CD21^+^ networks, TLSs formed reactive germinal centres as detected by peanut agglutinin (PNA) and Bcl‐6 staining in sequential tissue sections (Fig. 1 *e*), and the presence of Ki67^+^ proliferating cells characteristic of germinal centre B cells (Fig. 1 *f*). Interestingly, the TLSs observed were heterogeneous, ranging from dense aggregates of co‐localised B and T cells to compartmentalised TLSs displaying B and T cell segregation (Fig. 1 *g*). While this suggests the presence of TLSs with differing degree of maturity, immunohistochemical staining of sequential sections revealed that compartmentalised TLSs, as well as those lacking fully distinct B and T cell zones, developed reactive germinal centres (Fig. 1 *g*). These cellular features consistent with the presence of TLSs were also supported at the molecular level, whereby qPCR confirmed the expression of *Cxcl13*, and that of *Ccl19*, *Cxcl12* and *Ccl21* in laser microdissected submucosal lymphoid aggregates (Fig. 1 *h*). Thus, tumourigenesis in *gp130* ^F/F^ mice is associated with the presence of organised lymphoid‐rich aggregates within the submucosa of the gastric antrum. The cellular composition and gene expression pattern displayed by these regions closely resemble TLSs.

### TLS development during gastric tumourigenesis coincides with the temporal induction of homeostatic chemokines

The development of TLSs is highly disease‐ and context‐dependent,^12^ and while their formation is linked with a number of cancers, their precise contribution to inflammation‐driven tumourigenesis in GC is unknown. Since the spontaneous development of gastric adenomas in *gp130* ^F/F^ mice occurs by 6 weeks of age,^24^, ^25^ we investigated whether hyperactive gp130 signalling induced submucosal TLSs prior to the development of gastric adenomas by staining for the presence of TLSs in 4‐week‐, 3‐month‐ and 6‐month‐old mice.

**Figure 2:**
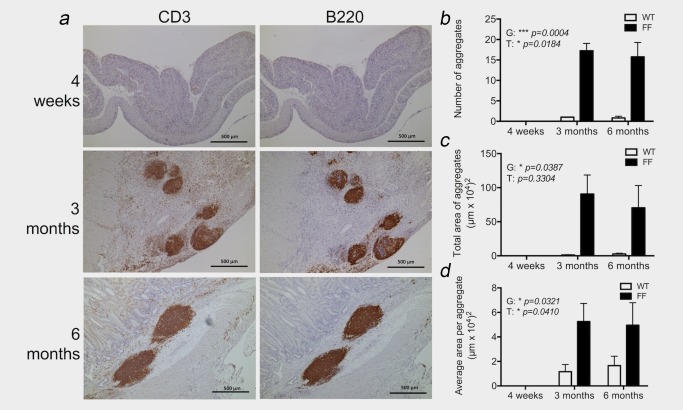
Temporal control of lymphoid neogenesis coincides with gastric tumour development in *gp130* ^F/F^ mice. (*a*) Representative immunohistochemistry of T cell (CD3) and B cell (B220) staining of lymphoid aggregates at time points representing pre (4‐week old), early (3‐month old) and advanced (6‐month old) gastric tumour development in *gp130* ^F/F^ mice. (*b* **–** *d*) The number (*b*), total area (*c*) and average size (*d*) of lymphoid aggregates were quantified in antral tissue cross‐sections of wild‐type (WT) and *gp130* ^F/F^ (FF) mice following immunohistochemical detection of B220 (*n* = 3/group at 4 weeks, *n* = 3/group at 3 months, *n* = 6–8/group at 6 months). Graphs represent mean ±SEM. *p*‐Values (*p < 0.05; ***p < 0.001) reveal a significant increase in the quantification of TLSs in the *gp130* ^F/F^ compared to wild‐type mice (G, genotype) by two‐way ANOVA. A significant increase in the quantification of TLSs is also observed over time (T, time point) between early (4 weeks) and late time points (3 and 6 months). Scale bars: 500 µm. [Color figure can be viewed at http://wileyonlinelibrary.com]

Immunohistochemistry for CD3 and B220 in 4‐week‐old *gp130* ^F/F^ mice that had not yet developed gastric adenomas revealed that TLSs were absent (Fig. 2 *a*). In older 3‐ and 6‐month‐old *gp130* ^F/F^ mice that had developed gastric tumours, submucosal TLSs were clearly identified as dense accumulations of T and B cells (Fig. 2 *a*). While B220^+^ B cells were mostly confined to lymphoid aggregates, T cells could also be detected as diffuse infiltrates throughout the gastric submucosa (Fig. 2 *a*). Given that B cells were most prominently associated with TLSs, lymphoid aggregates were quantified based on B220^+^ immunohistochemistry staining. Both the total number of lymphoid aggregates, the total area of submucosal TLSs and the average size of TLSs was significantly increased in *gp130* ^F/F^ mice compared to control WT mice (Figs. 2 *b*–2 *d*), with no differences in these parameters between 3‐ and 6‐month‐old *gp130* ^F/F^ mice. Thus, TLS development in response to hyperactive gp130 signalling is temporally linked with the establishment of gastric adenomas.

**Figure 3:**
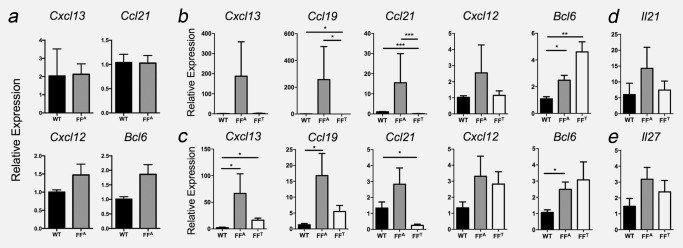
Tumour‐associated TLS development is accompanied with heightened expression of lymphoid chemokines. Temporal qPCR analysis of TLS‐associated genes in the gastric antrum of *gp130^+/+^* mice (WT) and *gp130* ^F/F^ mice (FF^A^). Relative gene expression is presented in mice without GC at 4 weeks (*a*), 3 months (*b*) and 6 months (*c*) of age. Expression of the cytokines *Il21* (*d*) and *Il27* (*e*) was also determined in the gastric antrum of 6‐month‐old *gp130* ^F/F^ and WT mice. In 3‐ and 6‐month‐old *gp130* ^F/F^ mice, gene expression was also determined in the gastric tumour tissue (FF^T^) (*n* = 4 per group at 4 weeks, *n* = 7 per group at 3 months, *n* = 6–7 per group at 6 months). Graphs represent mean ± SEM. *p < 0.05; **p < 0.01; ***p < 0.001.

Homeostatic chemokines play a central role in the development of secondary lymphoid organs and TLSs,^4^, ^12^ and our earlier data revealed transcripts for *Cxcl13*, *Ccl19*, *Ccl21* and *Cxcl12* in laser microdissected TLSs from 6‐month‐old *gp130^F/F^* mice with well‐established gastric tumours (see Fig. 1 *h*). To determine whether TLS development was associated with the temporal induction of key lymphoid chemokines, we quantified *Cxcl13*, *Ccl19*, *Ccl21* and *Cxcl12* in the gastric antrum of *gp130* ^F/F^ and control WT mice (Fig. 3). Notably, in 4‐week‐old *gp130* ^F/F^ mice the expression of *Cxcl13*, *Ccl21* and *Cxcl12* was comparable to WT mice (Fig. 3 *a*). Furthermore, at this early time point that preceded the development of gastric adenomas and submucosal TLSs, transcripts for the homeostatic chemokine *Ccl19* were not detected. Expression of *Bcl6*, the master transcriptional regulator of T follicular helper (Tfh) cells and germinal centre B cells, was also comparable between 4‐week‐old *gp130* ^F/F^ and WT mice (Fig. 3 *a*). In 3‐ and 6‐month‐old *gp130* ^F/F^ mice, TLS development was associated with increased expression of *Cxcl13*, *Ccl19* and *Ccl21* in the gastric antrum tissue (FF^A^; primarily comprising submucosal tissue) when compared to control WT antrum, or when expression was compared between the *gp130* ^F/F^ gastric antrum and mucosal tumour (FF^T^) tissues of these mice (Figs. 3 *b* and 3 *c*). Consistent with the detection of Bcl‐6 at tumour‐associated TLSs (see Fig. 1 *e*), *Bcl6* expression was increased in *gp130* ^F/F^ antrum tissue suggesting the presence of germinal centre B cells or Tfh cells, and was also highly expressed in the tumours of *gp130* ^F/F^ mice (Figs. 3 *b* and 3 *c*). However, no significant differences were observed for the expression of the Th17/Tfh effector cytokine, IL‐21 (Fig. 3 *d*). We recently described the cytokine IL‐27 as an inhibitor of synovial TLS development in inflammatory arthritis.^22^ The expression of *Il27*, which encodes for the IL‐27p28 subunit, was also comparable between WT and *gp130* ^F/F^ stomach antrum (Fig. 3 *e*), suggesting that TLS development in *gp130* ^F/F^ mice was not linked to a loss of an inhibitory IL‐27 signal. Collectively, these observations suggest that a temporal induction of homeostatic chemokines during gastric tumourigenesis drives the development and maintenance of TLSs.

**Figure 4:**
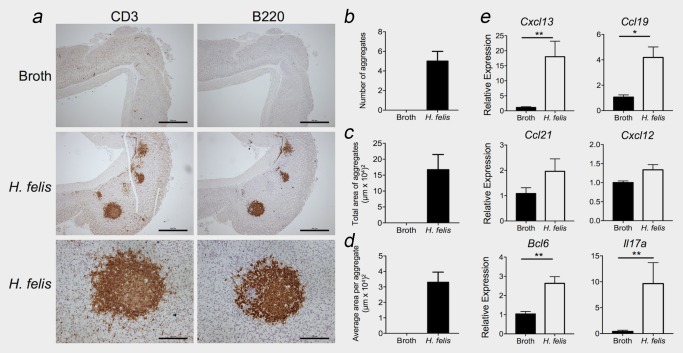
Chronic *Helicobacter* infection drives TLS development in the absence of gastric tumourigenesis. (*a*) Representative immunohistochemistry for CD3^+^ T cells and B220^+^ B cells in the gastric submucosa of WT mice intragastrically treated with BHI broth (top panel) or *H. felis* (middle and bottom panels). (*b–d*) The number (*b*), total area (*c*) and average size (*d*) of lymphoid aggregates were quantified in gastric tissue cross‐sections following immunohistochemistry for B220 (*n* = 3/group). (*e*) qPCR for the indicated genes was normalised to 18S rRNA in the gastric antrum of *H. felis‐*infected and control mice (*n* = 5/group). Graphs represent mean ± SEM. *p < 0.05; **p < 0.01. Scale bars: 500 µm top and middle panels, 125 µm bottom panel. [Color figure can be viewed at http://wileyonlinelibrary.com]

Given that chronic gastritis as a consequence of *H. pylori* infection is linked with the development of >75% of intestinal‐type GC cases,^36^ we also assessed formation of TLSs in a chronic *Helicobacter* infection model. Upon infection with *H. felis*, 100% of WT mice developed submucosal TLSs (Figs. 4 *a*–4 *d*). Consistent with the clinical presence of mucosal lymphoid aggregates and the expression of homeostatic chemokines in *H. pylori‐*infected individuals,^37^ TLS development in *H. felis‐*infected mice was associated with the heightened expression of *Cxcl13*, *Ccl19*, *Bcl6* and *Il17a* (Fig. 4 *e*). Thus, these data suggest that TLS formation is a feature of *Helicobacter* infection‐induced gastritis prior to the onset of gastric tumours.

### Activation of gp130/STAT3 signalling is essential for TLS development in *gp130* ^F/F^ mice, but lymphoid neogenesis is independent of IL‐17

**Figure 5:**
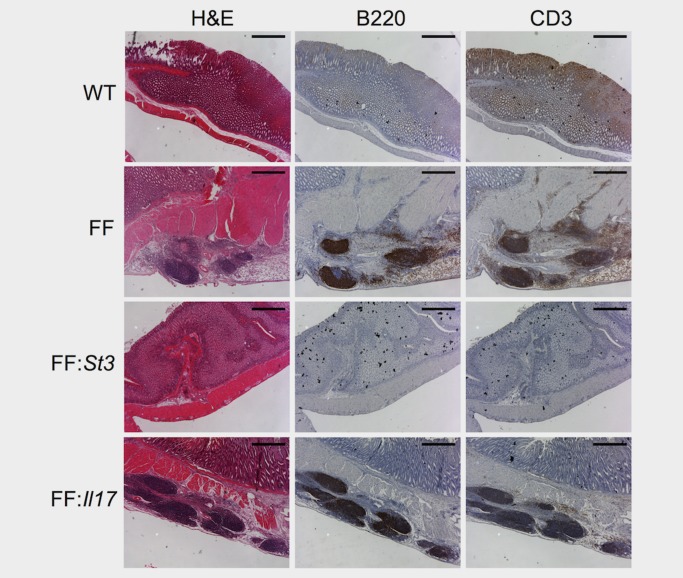
Development of tumour‐associated TLSs in *gp130* ^F/F^ mice is STAT3 driven but is independent of IL‐17. H&E and immunohistochemistry for B cells (B220) and T cells (CD3) in tumour‐associated TLSs of 6‐month‐old *gp130^+/+^* (WT), *gp130* ^F/F^ (FF), *gp130* ^F/F^ *:Stat3^+/^* ^−^ (FF:*St3*) and *gp130* ^F/F^ *:IL17a* ^−^^*/*^^−^ (FF:*Il17*) mice (representative images from *n* = 6 *gp130^+/+^*, *n* = 5 *gp130* ^F/F^, *n* = 4 *gp130* ^F/F^ *:IL17a* ^−^^*/*^^−^, *n* = 4 *gp130* ^F/F^ *:Stat3^+/^* ^−^). Scale bars: 500 µm. [Color figure can be viewed at http://wileyonlinelibrary.com]

Cytokines that activate STAT3 have previously been linked with TLS development and maintenance, including IL‐6, IL‐21 and IL‐22.^21^, ^23^, ^38^, ^39^ Consistent with a potential role for gp130‐mediated STAT3 activity in TLS development, immunohistochemistry of sections from 6‐month‐old *gp130* ^F/F^ mice revealed the presence of phosphorylated tyrosine (p‐Y)^705^STAT3^+^ cells in submucosal TLSs (Supporting Information Fig. S1). While p‐Y^705^STAT3 was detected at TLSs, STAT3 activity was not restricted to lymphoid aggregates but was also found in regions of diffuse lymphocyte infiltration. In light of the above observations, we genetically defined whether reducing endogenous STAT3 activity in *gp130* ^F/F^ mice, which we have previously shown abrogates the development of gastric adenomas,^25^ would influence submucosal TLS development. Indeed, the close association between gastric tumourigenesis and submucosal lymphoid neogenesis was further confirmed by the absence of TLSs in the gastric antrum of *gp130* ^F/F^:*Stat3*± mice (Fig. 5). Thus, the development of gastric adenomas in *gp130* ^F/F^ mice and the associated development of TLSs are tightly coupled, and are dependent on hyperactivation of STAT3 *via* gp130 signalling.

Effector Th17 cell responses and IL‐17 have emerged as mediators of lymphoid neogenesis in various chronic inflammatory or autoimmune models.^35^, ^40^, ^41^ Interestingly, analysis of non‐tumour tissue from GC patients in TCGA data sets revealed that *IL17A* expression correlated significantly with that of *CXCL13* and *CCL19* (Supporting Information Fig. S2). Given that gp130‐mediated STAT3 activity drives Th17 cell responses, and that we have previously described elevated expression of the Th17‐associated markers *Il17a*, *Il23* and *Rorγt* in the *gp130* ^F/F^ GC model,^30^ we determined whether *Il17a* was required for the development of tumour‐associated TLSs. Notably, the formation of TLSs remained intact following the genetic ablation of IL‐17 in *gp130* ^F/F^:*Il17a* ^−^^*/*^^−^ mice (Fig. 5 and Supporting Information Fig. S3). Therefore, the development of TLSs in the *gp130* ^F/F^ model of inflammation‐associated GC is not IL‐17 dependent.

### A chemokine gene signature linked with TLS development in *gp130* ^F/F^ mice is associated with advanced GC in patients

**Figure 6:**
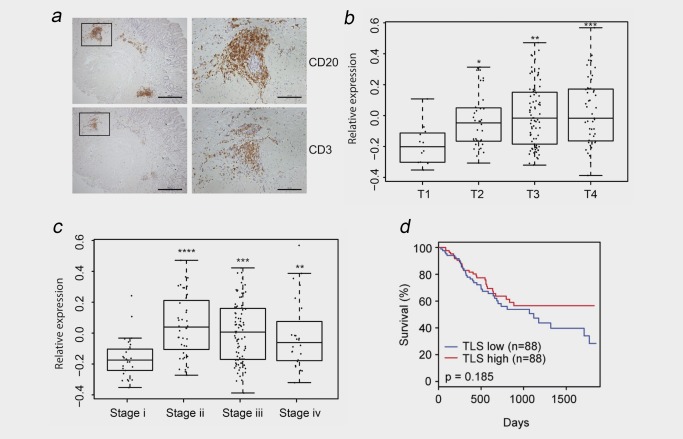
A TLS gene signature is associated with advanced GC in patients, but has no prognostic significance. (*a*) Representative immunohistochemistry for CD20^+^ B cells and CD3^+^ T cells in serial sections of human GC tissue biopsies showing the presence of TLSs in the tumoural submucosa (Scale bars: 500 µm left images; 125 µm right images). Sequential sections on the right show higher‐power images of a TLS (corresponding to the boxed regions in the left panel) displaying T and B cell segregation. (*b*–*d*) Evaluation of the expression of a TLS gene signature comprising *CXCL13*, *CCL19* and *CCL21* in TCGA data sets of 176 GC patients with intestinal‐type GC. An association between the TLS gene signature and tumour growth (*b*), the stage of GC (*c*) and patient survival (*d*) is presented. *p < 0.05; **p < 0.01; ***p < 0.001; ****p < 0.0001. Scale bars: 500 µm (left); 125 µm (right). [Color figure can be viewed at http://wileyonlinelibrary.com]

Gene signatures of TLS development or activity have shown prognostic value in certain cancers.^13^, ^14^, ^15^ We therefore evaluated whether lymphoid aggregates reminiscent of TLSs were present in biopsy samples from intestinal‐type GC patients. In all patients with adenocarcinomas, extra‐tumoural CD20^+^ B cell‐ and CD3^+^ T cell‐rich lymphoid aggregates were observed in the gastric submucosa (Fig. 6 *a* and Supporting Information Fig. S4). Consistent with the appearance of TLSs in the *gp130* ^F/F^ model of GC, TLSs in clinical disease ranged from aggregates of co‐localised B and T cells to TLSs displaying B and T cell segregation (Fig. 6 *a*, right panel). TLSs were not observed in gastritis patients with precursor lesions such as intestinal metaplasia.

To determine the clinicopathological relevance of TLSs in intestinal‐type human GC, we investigated the expression of a 3‐gene TLS signature comprising the homeostatic chemokines *CXCL13*, *CCL19* and *CCL21* (identified in the *gp130* ^F/F^ mouse model; see Fig. 3) in TCGA data sets (Figs. 6 *b*–6 *d*). Here, compared to patients where tumour growth was confined to the mucosa and submucosa (stage T1), increased expression of the 3‐gene signature was observed in patients where the tumour had invaded the subserosal layer, the serosa or nearby organs or structures (stages T2, T3 and T4) (Fig. 6 *b*). Consistent with the temporal development of TLSs during STAT3‐driven tumourigenesis in *gp130* ^F/F^ mice, the TLS gene signature was enriched in patients with more advanced stage GC (stages ii, iii and iv) (Fig. 6 *c*), and correlated with the expression of the STAT3 target gene, *SOCS3* (Supporting Information Fig. S5). While an association was observed between the expression of the *CXCL13/CCL19/CCL21* signature and tumour growth (T stage), no association was observed with lymph node involvement (N stage) or degree of metastasis (M stage) (Supporting Information Fig. S6). Similar trends were observed when the expression of *CXCL13*, *CCL19* and *CCL21* were individually assessed in TCGA data sets for intestinal‐type GC (Supporting Information Fig. S6). Finally, when we evaluated the expression of the TLS signature genes in relation to patient survival, no statistical significance was observed (Fig. 6 *d* and Supporting Information Fig. S6). Similarly, no statistical significance was observed when the TLS gene signature was evaluated in an independent Asian Cancer Research Group (ACRG) cohort of GC patients (GEO accession GSE62254; Supporting Information Fig. S7).^34^ Thus, mirroring the temporal development of TLSs in established disease in the gp130^F/F^ GC model, TLSs and an associated 3‐gene signature were also observed in human GC, albeit without prognostic value.

## Discussion

Over the last decade, an appreciation that TLSs are associated with cancers has resulted in an increasing interest in understanding their development and potential as immunomodulatory targets for enhancing anti‐tumour immunity. Histopathological evaluation of tumour biopsies has revealed a positive correlation between the frequency of tumour‐associated TLSs and patient survival in numerous cancers.^5^, ^6^, ^7^, ^8^, ^9^, ^10^, ^11^ It is therefore proposed that TLSs control the recruitment and induction of tumour‐specific T and B cell responses that can limit cancer progression.^4^, ^12^ While these studies emphasise the role of TLSs in anti‐tumour immunity, the mechanisms governing TLS development remain poorly defined. Using a clinically relevant mouse model of STAT3‐driven GC,^24^, ^25^, ^26^ we demonstrate that tumourigenesis coincides with the development of organised TLSs. Here, TLS formation was accompanied by the expression of homeostatic chemokines (e.g., *Cxcl13*, *Ccl19* and *Ccl21*) linked with the arrangement of lymphocytes, stromal cells and monocytic cells within lymphoid structures. Consistent with our previous studies that highlight submucosal inflammation as an ever‐present feature of gastric tumourigenesis,^24^, ^25^, ^26^ while TLS development and tumourigenesis were closely linked in *gp130* ^F/F^ mice, gastritis as a result of *Helicobacter* infection could also trigger lymphoid neogenesis.

Recently, a histological evaluation of GC patients revealed that the presence of peri‐tumoural CD20^+^ B cell aggregates was associated with improved relapse‐free survival.^20^ Thus, GC progression is influenced by the development and activity of immune cell networks within tumour‐associated TLSs. Detection of a TLS‐associated gene signature may therefore act as a reliable prognostic indicator of patient outcome. Notably, the expression of TLS‐related genes including *CXCL13*, *CCL19* and *CCL21*, has been linked with a good prognosis in colorectal cancer^14^, ^15^ and melanoma.^13^ Interestingly, expression of a 3‐gene TLS signature comprising *CXCL13*, *CCL19* and *CCL21* was increased in patients with advanced GC. Consistent with the heightened expression of *Il17a* and lymphoid chemokines in the *gp130* ^F/F^ mouse model of GC, *IL17A* expression also correlated with the 3‐gene TLS signature, *CXCL13* and *CCL19* in clinical disease. However, the 3‐gene TLS signature investigated herein did not predict favourable GC patient outcomes. Consistent with this observation, a B cell metagene signature investigated in a recent study of GC only had prognostic significance when combined with a Th1 gene signature.^20^ In the same study, the authors noted that while the histological presence of peri‐tumoural TLSs was linked with improved relapse‐free survival, greater prognostic value was observed when TLSs were associated with the presence of T‐bet^+^ tumour‐infiltrating lymphocytes marking a robust Th1 response.^20^ Thus, histopathological evaluation of TLS development may have better prognostic potential in GC than the use of B cell or TLS gene signatures. To better exploit the therapeutic potential of TLSs, further research is required to develop improved TLS gene signatures that can mark the presence of robust anti‐tumour responses.

Lymphokines that signal through gp130 are key regulators of TLSs in chronic inflammatory conditions. For example, transgenic overexpression of IL‐6 and the IL‐6 receptor (IL‐6R), which results in potent STAT3 activation, drives CXCL13 production and TLS development in the lung.^21^ The IL‐6 related cytokine, IL‐27, also utilises gp130 for signalling. However while IL‐6 is primarily pro‐inflammatory, IL‐27 can preferentially activate STAT1 to exert suppressive effects on adaptive immune cells.^42^ For example, IL‐27 inhibits effector T cell responses, counteracts the IL‐6/STAT3 driven differentiation of Th17 cells and suppresses TLS development.^22^, ^42^, ^43^ Our analysis of gastric antrum in WT and *gp130* ^F/F^ mice revealed a comparable expression of *Il27*, suggesting that the development of tumour‐associated TLSs was independent of any inhibitory action potentially imposed by IL‐27. The studies outlined above suggest that gp130‐mediated STAT3 activation may be a key determinant of TLS development in inflamed tissues. Consistent with this, both TLS development and gastric tumourigenesis in *gp130* ^F/F^ mice were closely linked, and were dependent on hyperactive STAT3 activity. Normalisation of STAT3 levels in *gp130* ^F/F^:*Stat3*± mice inhibited both tumour development and lymphoid neogenesis. Notably, while STAT3 phosphorylation was clearly detected in TLSs, STAT3 activation was not confined to these sites but was also found in areas of diffuse lymphocyte infiltration. Therefore, despite a role for STAT3 activating cytokines in lymphoid neogenesis,^4^, ^21^, ^23^, ^38^, ^39^ localised STAT3 activity remains a poor indicator of TLS involvement.

Many of the above STAT3‐activating cytokines are linked with the development, maintenance or effector function of Th17 cells.^44^ This T cell subset, and its signature cytokine IL‐17, has been associated with TLS development in infection, autoimmunity, allograft rejection and cancer.^4^, ^35^, ^39^, ^40^, ^41^ Nevertheless, a role for Th17/IL‐17 in TLS development remains controversial and is likely disease‐ and context‐dependent. For example, IL‐17 drives the development of TLSs (called induced bronchus associated lymphoid tissue; iBALT) in the lungs of neonatal mice challenged with lipopolysaccharide.^41^ Comparable studies show that the formation of iBALT in response to *Pseudomonas aeruginosa* infection is IL‐17 dependent.^40^ However, the development of iBALT following infection with modified vaccinia virus Ankara was unimpaired in the absence of IL‐17.^40^, ^45^ A similar context‐dependent role for IL‐17 in TLS development is observed in models of autoimmunity. Th17 cells and IL‐17 play a prominent role in the development of TLSs during experimental autoimmune encephalomyelitis,^35^ but are not central to lymphoid neogenesis in inflamed salivary glands.^38^ Our data illustrates that while GC development in *gp130* ^F/F^ mice is linked with the heightened expression of the Th17‐associated markers *Il17a*, *Il23* and *Rorγt*,^30^ the signature Th17 cytokine IL‐17 is not required for gastric tumourigenesis or the development of submucosal TLSs. Nevertheless, other cytokines linked with the Th17 programme may contribute to lymphoid neogenesis in GC. For example, IL‐22 and IL‐23 have emerged as drivers of lymphoid neogenesis in other inflammatory settings,^4^, ^35^, ^38^ and are linked with human GC progression.^30^, ^46^, ^47^

Studies have demonstrated that coordinated B cell and Th1 cell responses at TLSs favour improved patient outcomes in GC and non‐small cell lung carcinoma.^7^, ^20^ While follicular B cells within TLSs support humoral immunity, and can generate antibodies that recognise tumour antigens,^8^ they also provide costimulatory signals that drive T cell priming and expansion.^48^ Thus, an environment that favours Th1‐type responses at TLSs, as has been described in other types of cancer,^5^, ^7^, ^14^, ^49^ may be required to sustain anti‐tumour responses that favour improved patient outcomes. While the tumour‐associated TLSs observed in *gp130* ^F/F^ mice display B and T cell compartmentalisation and germinal centre reactions, the failure to mount robust anti‐tumour responses that prevent tumourigenesis may relate to the location of TLSs. Tumour‐associated TLSs were observed in the tumoural and gastric submucosa of *gp130* ^F/F^ mice and humans with GC, but were not seen in the tumour stroma. High densities of intra‐tumoural TLSs and HEV have been linked with enhanced local Th1 responses and improved patient survival.^6^, ^50^ By contrast, and consistent with the location of TLSs in our study of GC, the presence of extra‐tumoural TLSs in colorectal cancer did not yield a positive prognosis but reflected a pro‐inflammatory microenvironment surrounding tumours in advanced disease.^18^ Therefore, in some cancers extra‐tumoural TLSs may reflect a pathological consequence of inflammation‐associated tumourigenesis, rather than markers of an effective anti‐cancer response. Further studies are thus required to better define the relationship between the location of TLS development and patient prognosis. In a previous study of TLSs in GC, a B cell gene signature representative of TLSs only provided prognostic value when combined with a robust Th1 gene signature.^20^ Therefore, in certain cancers, TLSs may mark an unsuccessful attempt to establish anti‐tumour immunity, and in some cases even provide immunopathological microniches that support cancer progression.^19^

In summary, our data show that gastric tumourigenesis as a consequence of hyperactive gp130/STAT3 signalling, is intrinsically linked with the development of submucosal TLSs in gastric antrum tissue. A TLS gene signature was associated with advanced disease in GC patients, but did not indicate favourable prognosis. Given that TLSs and associated Th1 responses have emerged as predictors of improved patient outcomes in certain cancers, further translational studies are needed to define robust context‐dependent biomarkers of TLS activity. Such insight offers the opportunity to better understand the role of TLSs in GC progression as a route to improved patient diagnosis and treatment.

## Author Contributions

GWJ and BJJ designed the study and wrote the manuscript. DGH, LM, GWJ and AG contributed to the histopathological characterisation of lymphoid aggregates in gastric tissue. AW, JJB, HO, MO and LM analysed gastric tissue gene expression by qPCR, and HO and MO performed laser microdissection. LY and HG performed bioinformatics analysis using The Cancer Genome Atlas data sets. The mouse model of chronic gastritis induced by *Helicobacter* infection was performed by JJB, KD and RLF. PSB and WS provided human gastric tissue biopsies and pathological assessments.

## Supporting information

Supporting Information
