# Supplementary material for: Hyperactive gp130/STAT3‐driven gastric tumourigenesis promotes submucosal tertiary lymphoid structure development
Source: Int J Cancer. 2018 Feb 19;143(1):167–78. doi: 10.1002/ijc.31298 (PMC5969244; doi:10.1002/ijc.31298)
Supplement: Supplementary file 1 — Supporting Information [file IJC-143-167-s001.pdf]

Supplementary Material. *Hill et al, Hyperactive gp130/STAT3-driven gastric tumourigenesis promotes submucosal tertiary lymphoid structure development*

Table S1. Primer sequences for SYBR green-based qPCR and PCR amplification of the *H.felis flaB* gene.

**Name**	**Sequence**
Cxcl13 forward	5’-TGCCCCAAAACTGAAGTTGTGATCT-3’
Cxcl13 reverse	5’-ACTCACTGGAGCTTGGGGAGTTGA-3’
Ccl19 forward	5’-GCGCACACAGTCTCTCAGGCTC-3’
Ccl19 reverse	5’-AGTTGGGGCTGGGAAGGTCCA-3’
Ccl21 forward	5’-CCCCTGGACCCAAGGCAGTGA-3’
Ccl21 reverse	5’-TTGCCGGGATGGGACAGCCT-3’
Cxcl12 forward	5’-GCGCTCTGCATCAGTGACGGTAA-3’
Cxcl12 reverse	5’-GCTTGACGTTGGCTCTGGCGA-3’
Bcl6 forward	5’-CCTGCAACTGGAAGAAGTATAAG-3’
Bcl6 reverse	5’-AGTATGGAGGCACATCTCTGTAT-3’
Il21 forward	5’-CCCCAAGGGCCAGATCGCCT-3’
Il21 reverse	5’-TGCATGCTCACAGTGCCCCTTT-3’
Il27 forward	5’-CGATTGCCAGGAGTGAACCT-3’
Il27 reverse	5’-CAGAGTCAGAGAGGTGATGCC-3’
Il17a forward	5′-ACCGCAATGAAGACCCTGAT-3′
Il17a reverse	5′-TCCCTCCGCATTGACACA-3′
18S rRNA forward	5’-GTAACCCGTTGAACCCCATT-3’
18S rRNA reverse	5’-CCATCCAATCGGTAGTAGCG-3’
H. felis flaB forward	5'-TTCGATTGGTCCTACAGGCTCAGA-3'
H. felis flaB reverse	5'-TTCTTGTTGATGACATTGACCAACGCA-3'

Table S2. Clinicopathological features and demographics of gastric cancer patient cohorts from The Cancer Genome Atlas (TCGA) used for expression profiling of ELS.

	TCGA cohort (Intestinal-type)	TCGA cohort (Normal tissue)
**Mean age**		
Years (range)	65.9 (30-90)	67 (46-88)
**Gender**[a]		
Male	116 (66)	23 (66)
Female	60 (34)	12 (34)
Unknown	0 (0)	0 (0)
**Lauren class**[a]		
Intestinal-type	176 (100)	13 (37)
Diffuse	0 (0)	6 (17)
Unknown	0 (0)	16 (46)
***H. pylori* infection**[a]		
Positive	12 (7)	1 (3)
Negative	101 (57)	7 (20)
Unknown	63 (36)	27 (77)
**Tumour grade**[a]		
1	6 (3)	0 (0)
2	92 (52)	14 (40)
3	75 (43)	21 (60)
4	0 (0)	0 (0)
Unknown[b]	3 (2)	0 (0)

[a]Values in parentheses are %.

[b]No information available.

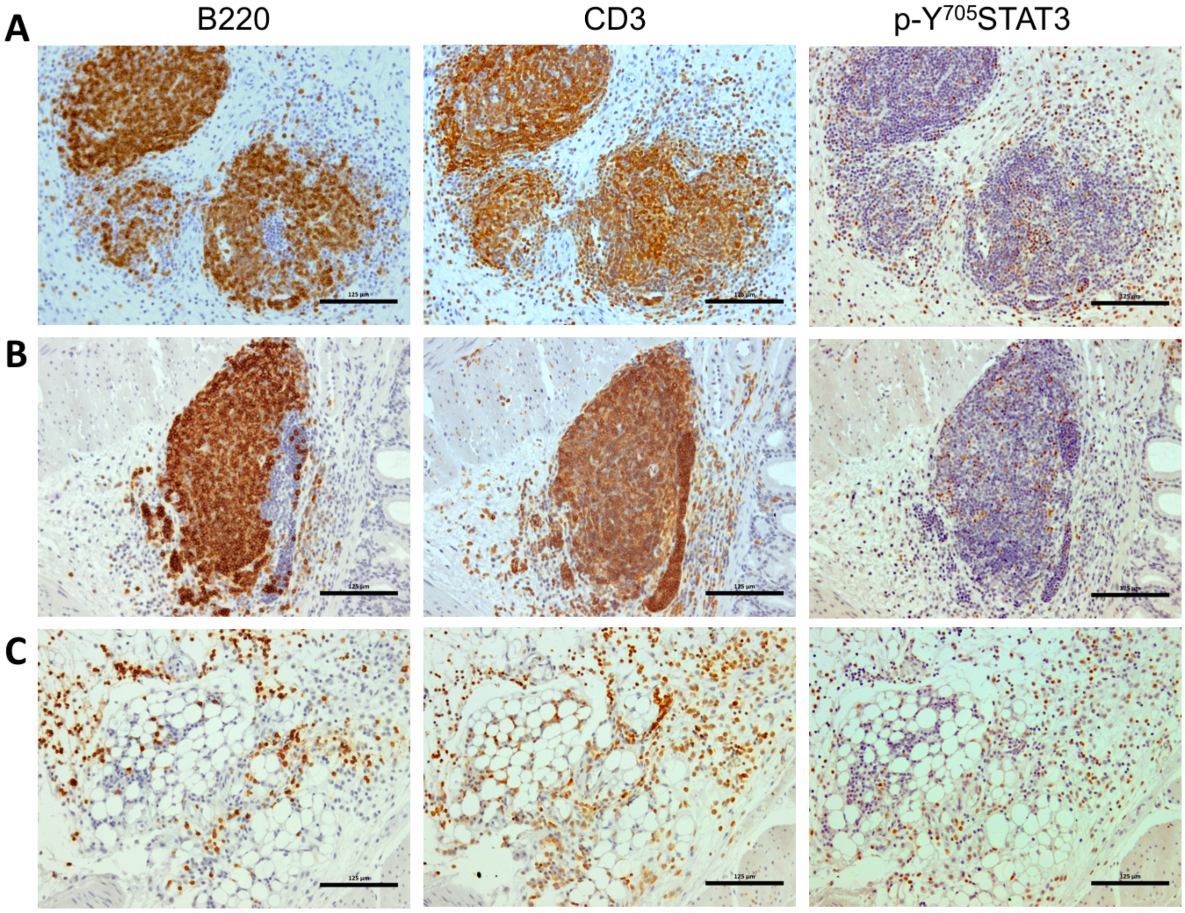

Figure S1. Immunohistochemistry for p-Y^{705}STAT3 at submucosal tumour-associated TLS in *gp130*$^{F/F}$ mice. Representative immunohistochemistry of serial gastric antrum sections for B220, CD3 and p-Y^{705}STAT3 in 3-month-old (**A**) and 6-month-old (**B-C**) *gp130*$^{F/F}$ mice. Cells positive for p-Y^{705}STAT3 can be seen in lymphoid aggregates (**A-B**) and in diffuse areas of inflammation (**C**). Scale bar: 125 µm.

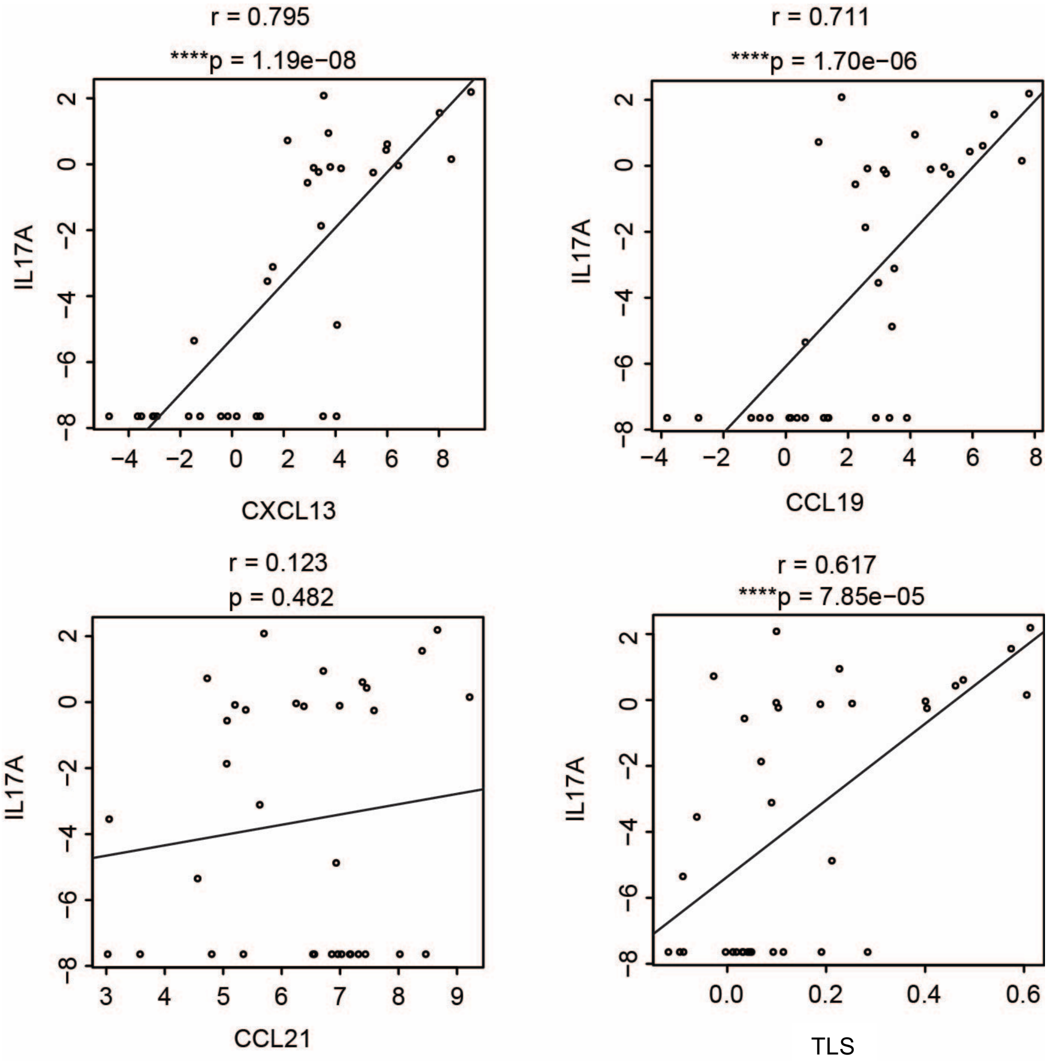

Figure S2. Correlations between *IL17A* and lymphoid chemokine expression in human gastric cancer (GC). Analysis by Spearman correlation coefficients of the gene expression between *IL17A* and *CXCL13*, *CCL19*, *CCL21* and a 3-gene TLS signature in 35 matched non-tumour (NT) tissues from The Cancer Genome Atlas GC cohort. The 3-gene TLS signature comprising *CXCL13*, *CCL19* and *CCL21* was identified in the *gp130*$^{F/F}$ GC mouse model associated with submucosal ELS.

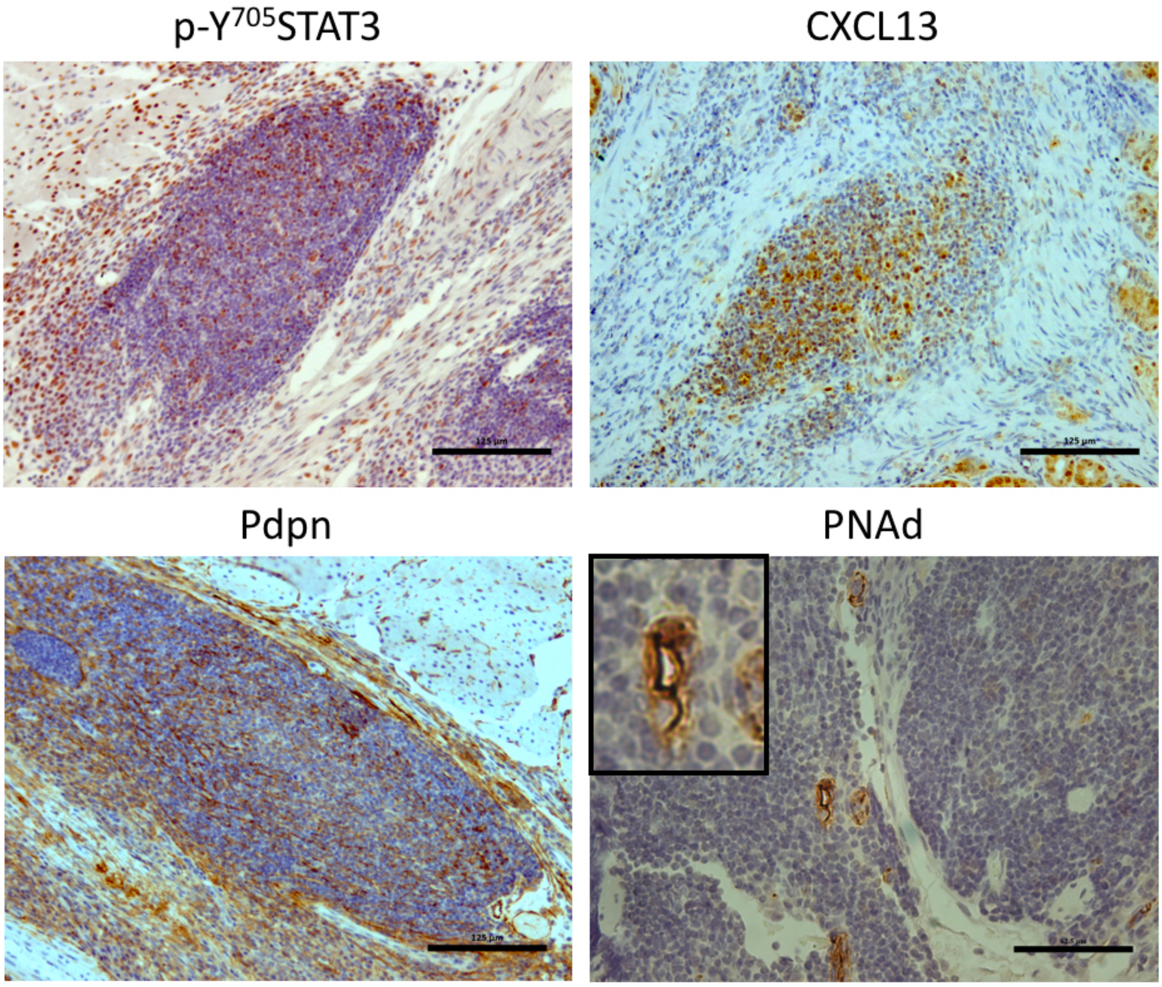

Figure S3. Characterisation of tumour-associated TLS in *gp130*$^{F/F}$*:Il17a*$^{-/-}$ mice. Representative IHC of p-Y^{705}STAT3, CXCL13, Pdpn and PNAd^{+} HEV at submucosal TLS in 6-month-old *gp130*$^{F/F}$*:Il17a*$^{-/-}$ mice. Scale bars: 125 µm.

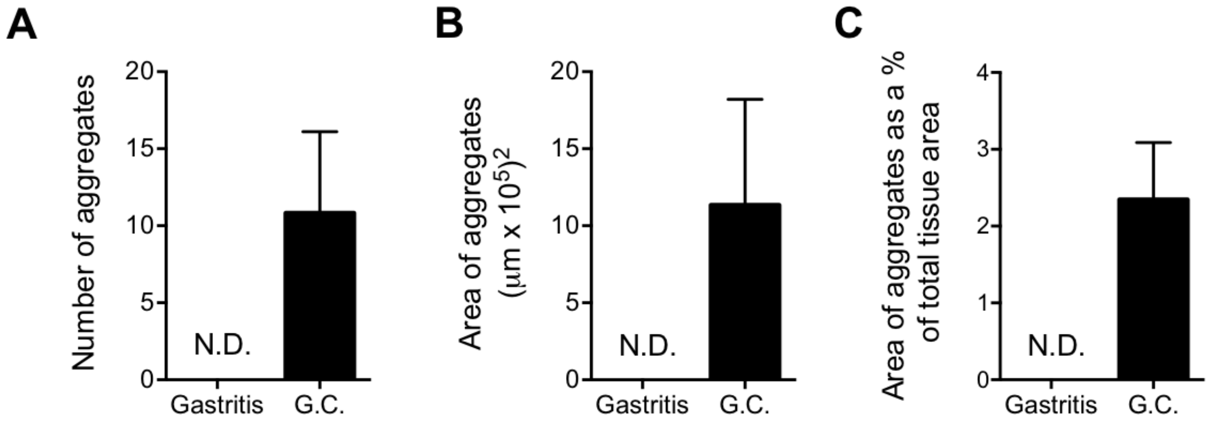

Figure S4. Quantification of TLSs in biopsy samples from patients with intestinal-type gastric cancer (GC). The number of TLSs (**A**), the total area of TLSs (**B**) and the area of TLSs as a percentage of total tissue area (**C**) was quantified in human GC tissue biopsies following immunohistochemical detection of $CD20^+$ cellular aggregates (n = 6). TLSs were absent in biopsies from patients with gastritis and intestinal metaplasia (n = 10). Graphs represent mean ±SEM. (N.D. None detected).

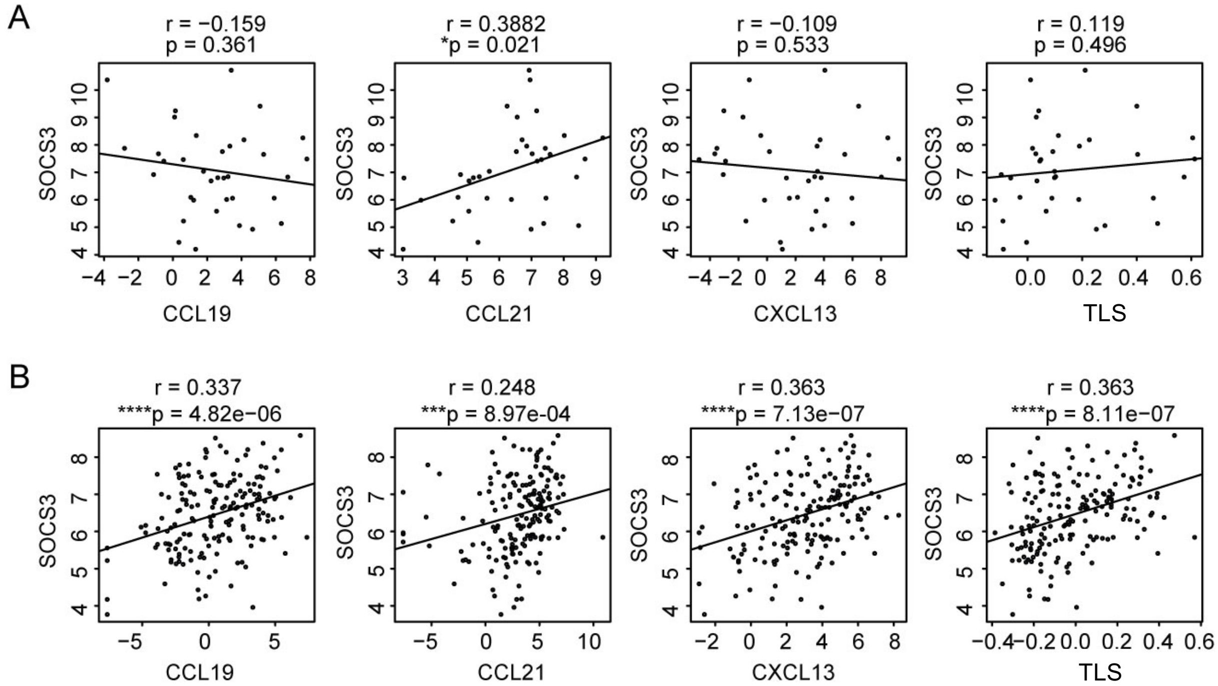

Figure S5. Correlations between the STAT3 target gene *SOCS3* and TLS related genes in human gastric cancer (GC). Analysis by Spearman correlation coefficients of the gene expression between *SOCS3* and *CCL19*, *CCL21*, *CXCL13* and the 3-gene TLS signature in 35 matched non-tumour tissues (**A**) and 176 intestinal-type tumour tissues (**B**) from The Cancer Genome Atlas GC cohort.

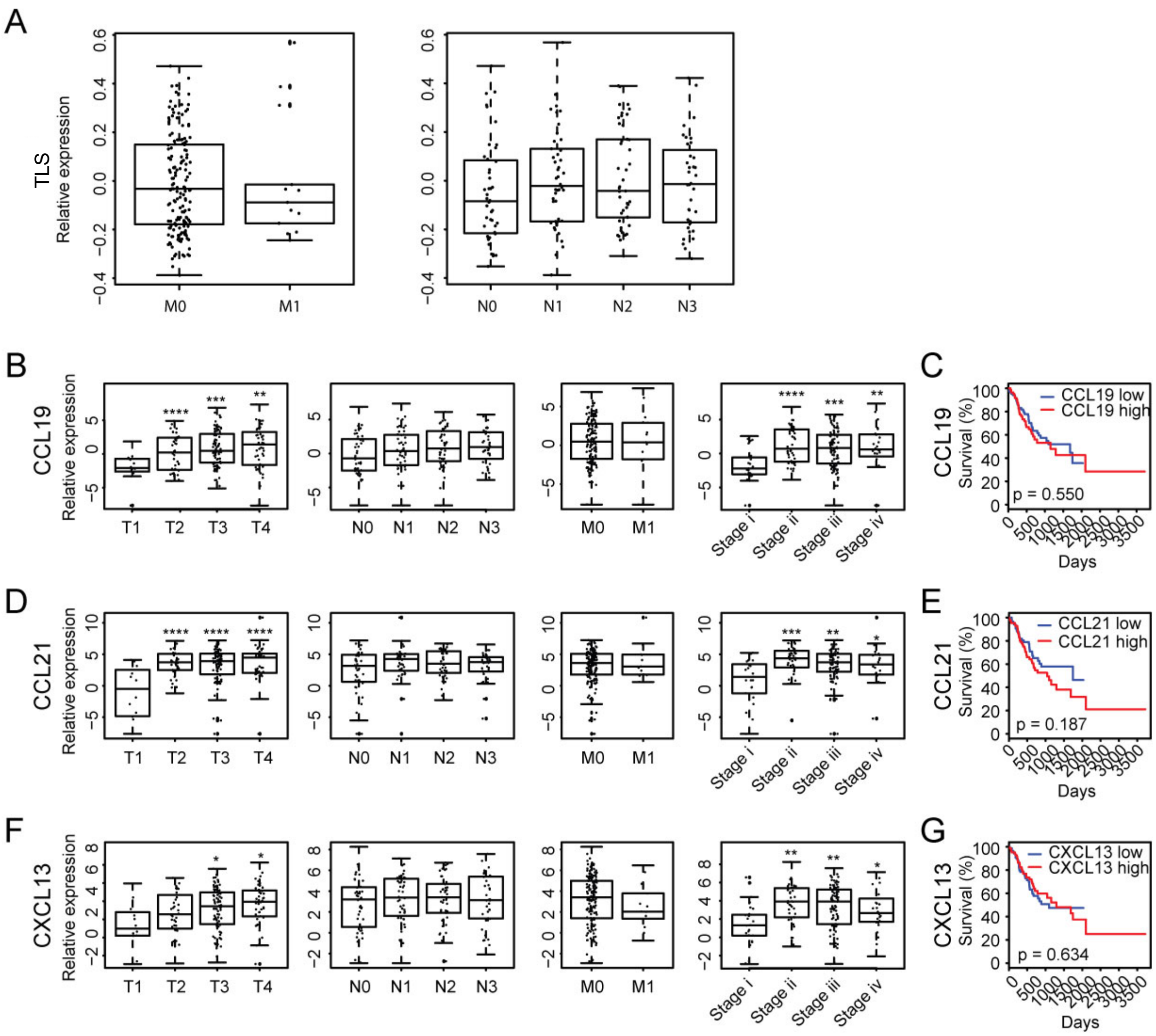

Figure S6. The association of TNM stage with the expression of a TLS gene signature and individual lymphoid chemokines in intestinal-type gastric cancer (GC). (**A**) Box and whisker plots showing the relative expression (logRPKM) of the 3-gene TLS signature at the indicated N and M stage of disease in The Cancer Genome Atlas (TCGA) cohorts with intestinal-type GC. (**B, D, F**) Box and whisker plots showing the relative expression of individual TLS genes (*CCL19*, *CCL21* and *CXCL13*) at the indicated TNM stage in TCGA cohorts with intestinal-type GC. (**C, E, G**) Kaplan-Meier overall survival curves for individual TLS genes with low and high expression in TCGA intestinal-type GC patients. Log rank with p-values were calculated. In B, D, F, data are presented as the mean ±SEM. *p<0.05, **p<0.01,

***p<0.001, ****p<0.0001. Detailed clinical information for TCGA patients is listed in Supplementary Table S2.

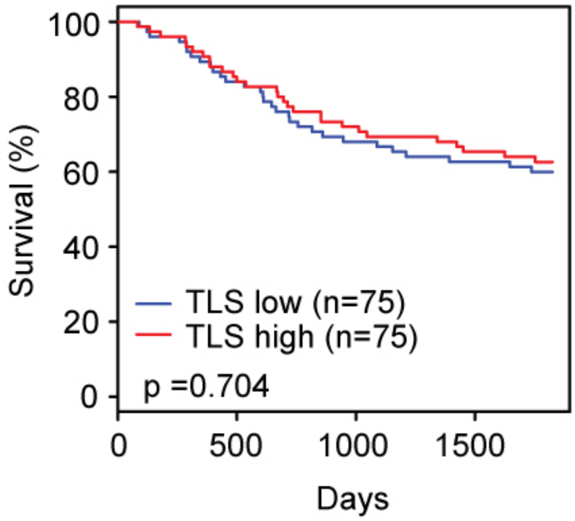

Figure S7. Evaluation of a TLS gene signature comprising *CXCL13, CCL19* and *CCL21* in an independent data set of intestinal-type gastric cancer (GC) patients from the Asian Cancer Research Group (ACRG) cohort. Patients were stratified into low or high expression of a TLS gene signature comprising *CXCL13*, *CCL19* and *CCL21* ('TLS high' or 'TLS low') by the median of RPKM in 150 intestinal-type GC patients in the ACRG cohort with follow-up information. Kaplan-Meier overall survival curves for subgroups with 'TLS high' and 'TLS low' are presented. Log rank with p-values were calculated.
